# The NHS breast cancer screening programme

**DOI:** 10.1038/sj.bjc.6601129

**Published:** 2003-07-01

**Authors:** N E Day

**Affiliations:** 1 Strangeways Research Laboratory, The Institute of Public Health, Wort's Causeway, Cambridge CB1 8RN, UK

Threlfall *et al* in this issue present direct evidence, for the first time, that the UK breast screening programme (NHSBSP) is reducing breast cancer mortality. As such it makes a valuable addition to the less direct approaches: one adopted by Blanks *et al* (2001) in their analysis of the different contributions to the decline in breast cancer mortality seen nationally, and another by McCann *et al* (2001) in their modelling of the likely impact on future breast cancer mortality of the change brought about by the NHSBSP in prognostic characteristics of breast cancer recorded at diagnosis. Viewed internationally, it adds to the results from Sweden (Tabar *et al*, 2003) and The Netherlands (Otto *et al*, 2003), which together have provided a powerful recent demonstration that routine mammography screening has reduced population breast cancer mortality rates. Taken together, these results confirm the conclusion from the Swedish randomized trials (World Health Organization. International Agency for Research on Cancer, 2002) that mammographic screening is an effective means of reducing breast cancer mortality, and show that the results from the more intensive setting of a randomized trial can be replicated in the service setting.

Besides documenting a decline in breast cancer mortality, the Threlfall *et al* paper shows that the degree of benefit from a breast screening programme is highly dependent on its quality. This, in turn, is determined by the degree of population coverage achieved and the quality of the screening. The latter is a complex issue, but two relatively simple closely related measures are the detection rate of small cancers (less than 10 or 15 mm in diameter) and the rate of interval cancers in each of the 3 years in the interscreening interval, both expressed as a multiple of the underlying breast cancer incidence rates. The authors focus on the first of these measures. The higher screening uptake rates in Wigan than in Manchester are reflected in greater reduction both in breast cancer mortality and in the incidence of advanced cancers. The difference, however, in the mortality rate reduction between the two programmes is more than could reasonably be attributed to the difference in uptake, assuming that uptake and mortality reduction are linearly related. There is also a greater detection rate of breast cancer at screening in the Wigan programme, particularly of small cancers. High sensitivity for the detection of small cancers is crucial, since the mortality reduction by screening is a consequence of diagnosing cancers earlier, and hence when smaller, survival improving by more than the lead-time associated with screening. In the first years of the NHSBSP, emphasis was placed on avoiding harm from screening, using easily measurable indicators such as the rate of recall for further assessment or the number of negative biopsies performed. Important as the avoidance of harm is, breast screening is pointless, even unethical, if the primary aim of detecting small cancers is not met. The recent results point clearly to the basic breast screening equation relating the mortality reduction to the extent to which diagnosis is brought forward in time.

Three years ago, discussion of this subject was hijacked by a review of the published trials, purporting to demonstrate that the major trials demonstrating the efficacy of breast screening were seriously flawed (Gotzsche and Olsen, 2000), later given the status of a Cochrane review (Olsen and Gotzsche, 2001). Major shortcomings in this review were clearly described, in the authoritative assessment of breast screening provided by a WHO workshop (World Health Organization. International Agency for Research on Cancer, 2002). It is unfortunate that the organization responsible for Cochrane reviews made no attempt to redress the damage done by this flawed review. The results of Threlfall *et al*, together with the Swedish work, now move the position forward. The question is not whether breast screening works, but how best to ensure that the benefits of a population screening programme can be brought up to the level of benefit achieved in the trials. This requires constant monitoring. Mammography screening may never make the major impact on breast cancer mortality that cervical cytology screening has made on cervical cancer mortality. It has the potential, however, to make a substantial reduction in breast cancer mortality, a potential now clearly being realised. Together with widespread dissemination of improved treatment modalities and greater awareness of the benefits of early diagnosis, the NHSBSP should make an increasing contribution in the next few years to reduction in breast cancer deaths, continuing the marked decline in breast cancer mortality seen over the past decade.
